# Oestradiol promotes the intrahepatic bile duct development of C57BL/6CrSlc mice during embryonic period via Notch signalling pathway

**DOI:** 10.1111/jcmm.16888

**Published:** 2021-09-08

**Authors:** Chen Dong, Ben‐ping Zhang, Yan‐Qin Ying, Ling Hou, Wei Wu, Hong Wei, Xiao‐ping Luo

**Affiliations:** ^1^ Department of Pediatrics Tongji Hospital Tongji Medical College Huazhong University of Science and Technology Wuhan China; ^2^ Department of Endocrinology Tongji Hospital Tongji Medical College Huazhong University of Science and Technology Wuhan China

**Keywords:** bile duct, cholangiocyte, hepatoblast, Notch signalling pathway, oestradiol

## Abstract

Oestradiol (E2) is a critical factor for multiple systems' development during the embryonic period. Here, we aimed to investigate the effects of oestradiol on intrahepatic bile duct development, which may allow a better understanding of congenital bile duct dysplasia. DLK^+^ hepatoblasts were extracted from the C57BL/6CrSlc foetal mice and randomly divided into control group, oestradiol groups (1, 10, 100 nM) and oestradiol (10 nM) + DAPT (inhibitor of Notch signalling; 40 µM) group for in vitro experiments. For in vivo analysis, pregnant mice were divided into control group, oestradiol (intraperitoneal injection of 0.6 mg/kg/day) ± DAPT (subcutaneous injection of 10 mg/kg/day) groups and tamoxifen (gavage administration of 0.4 mg/kg/day) group. The results showed that oestradiol promoted hepatoblast differentiation into cholangiocytes and intrahepatic bile duct development during the embryonic period. Tamoxifen, an antioestrogenic drug, inhibited the above processes. Moreover, oestradiol promoted the expression of Notch signalling pathway‐associated proteins and genes both in vitro and in vivo. Notably, DAPT addition inhibited the oestradiol‐mediated effects. In conclusion, oestradiol can promote hepatoblast differentiation into cholangiocytes and intrahepatic bile duct development of C57BL/6CrSlc mice during embryonic period via the Notch signalling pathway.

## 1 INTRODUCTION

Hepatocytes and cholangiocytes are the two major cells in the liver. Cholangiocytes can be further divided into two types: extrahepatic and intrahepatic cholangiocytes. Unlike the extrahepatic cholangiocytes, the intrahepatic cholangiocytes originate from bipotential hepatoblasts, which can also differentiate into hepatocytes during the embryonic period.^1^

The differentiation of hepatoblasts is regulated by different signalling pathways, especially by the Notch signalling pathway.^2^, ^3^ The activation of the pathway promotes hepatoblast differentiation into intrahepatic cholangiocytes and development of the intrahepatic bile ducts.^4^, ^5^, ^6^ Moreover, animal studies found that excessive activation of Notch signalling pathway causes hyperplasia, while the inhibition of the pathway causes dysplasia of the intrahepatic bile ducts in mice.^7^ Therefore, it is meaningful to study the factors influencing the Notch signalling pathway, so that to provide a basis for the prevention and treatment of intrahepatic biliary diseases.

Besides the close connection between Notch signalling pathway and intrahepatic bile ducts, oestradiol has been considered for many years to act as a major role in the development of the biliary tree.^8^ It acts in concert with several growth factors and modulates the proliferative activity of intrahepatic cholangiocytes.^9^, ^10^ In the bile duct ligated rat models, oestradiol prevents the cholangiocytes apoptosis and promotes the cholangiocyes proliferation. In the above process, the intracellular transduction pathways such as ERK and PI3K/AKT were reported the key points, while the Notch signalling pathway had not been mentioned.^11^, ^12^

It is worth noting that in breast cancer cells, endothelial cells and endometrial cancer cells, oestradiol has been reported to activate the Notch signalling pathway, thereby increasing the levels of receptors or legends by sixfold to eightfold.^3^, ^4^, ^13^, ^14^ Oestradiol is an important modulator of both foetal and maternal physiology during pregnancy.^15^, ^16^ Properly elevated blood level of oestradiol is important during pregnancy, with abnormally decreased levels observed in some pregnant women as a result of illness, certain medications or environmental reasons.^17^, ^18^ Thus, we hypothesized that for the development of intrahepatic bile ducts, there may be an interaction between oestradiol and Notch signalling pathway.

This study was aimed to demonstrate whether oestradiol is involved in the hepatoblast differentiation and intrahepatic bile duct development through the Notch signalling pathway.

## 2 MATERIALS AND METHODS

### 2.1 Mice

C57BL/6CrSlc mice of SPF grade were purchased from the experimental animal centre of China Three Gorges University. All mice were treated humanely, and the experimental protocols related to mice surgical operations were performed according to the guidelines of the Chinese Council on Animal Care and were approved by the Ethics Committee of Hospital, Tongji Medical College, Huazhong University of Science and Technology. The age of the embryos was determined according to the appearance of the vaginal plug. The day that the vaginal appeared was defined as 0.5 days of gestation.

### 2.2 Hepatoblast isolation and culture

The isolation of hepatoblasts and the cell cultures were performed as previously described.^19^ Briefly, after the pregnant C57BL/6CrSlc mice (13.5 days) were sacrificed by cervical dislocation, the livers of the foetal mice were carefully isolated from the uterus. Then, the liver tissues were minced and digested by type II collagenase solution/EDTA (1 g/L). After filtration and centrifugation, the cells were collected and resuspended in Dulbecco's modified Eagle's medium for adherent cultivation. The cell suspension was collected, washed and resuspended in phosphate‐buffered saline (PBS), containing 0.5% bovine serum albumin after twice adherent cultivations. For surface antigen determination, the cells were incubated with a rabbit anti‐DLK1 antibody (Abcam), washed twice in PBS and collected for flow cytometric analysis (Becton Dickinson). The cultures were maintained in a 37°C incubator, and the culture media was replaced every 2~3 days.

### 2.3 Flow cytometry

On day 13.5 of pregnancy, the liver of the foetal mice was isolated for the collection of hepatoblasts. The isolated hepatoblasts were inoculated into a 6‐well plate and incubated with various concentrations of oestradiol (1, 10 and 100 nM), with or without the addition of DAPT (an inhibitor of Notch signalling pathway; 40 μM) for 24 h. The cells were prepared by trypsin/EDTA and then collected, washed with PBS and pre‐cooling flow cytometry staining buffer, respectively. The cells were then incubated for 30 min with fixation/permeabilization buffer in the dark. After the addition of rabbit anti‐Sox9 and anti‐OPN antibodies (Sox, eBioscience; OPN, R&D), the cells were incubated for 30 min in the dark at 4°C. The collected cells were washed with permeabilization buffer twice and were analysed with a flow cytometer (Becton Dickinson) for detecting the surface antigens Sox9 and OPN after the addition of 200 μl of flow cytometry staining buffer.

### 2.4 Immunofluorescence analysis

Immunofluorescence analysis of the Notch signalling pathway and cholangiocyte lineage‐associated proteins in the cells was performed. The hepatoblasts derived from an E13.5 mouse were inoculated with oestradiol (1, 10 and 100 nM) or 10 nM oestradiol + 40 μM DAPT for 24 h. The slides covered with cells were washed with PBS for 3 times, followed by fixation with 4% paraformaldehyde for 15 min. Then, the cells were washed with PBS for another 3 times. After being blocked with goat serum for 30 min, the cells were, respectively, incubated with the primary antibodies at 4°C overnight. Then, the primary antibodies were detected with goat anti‐rabbit IgG (diluted 1:100; Boster) at 37°C for 60 min after three washes in PBS. The nuclei were incubated with DAPI for 5 min away from light, and the sections were examined using fluorescence microscope. Fluorescence quantification was achieved by comparing the integrated optical density/pixel value of the proteins in different group using Imagepro (ipp6.0).

### 2.5 RNA extraction and quantitative real‐time PCR (qRT‐PCR)

To quantify the mRNA expression of the proteins, RNA extraction was performed both in vitro and in vivo, using TRIzol reagent (Aidlab) according to the manufacturer's instructions. RNA was separated from the water phase by precipitation with isopropanol. The reaction mix of first‐strand cDNA synthesis is listed in Appendix Table A1. Total RNA (2 μg) was reverse‐transcribed to cDNA after treatment with DNase I (GeneCopoeia). The synthesis was incubated for 5 min at 25°C, followed by 15 min at 50°C, 5 min at 85°C and 10 min at 4°C. qRT‐PCR was performed as described previously.^20^ The cDNA was diluted 1:8 with nucleotide/RNAse free water. The reaction mix is listed in Appendix Table A2. The thermal cycle started with incubation for 2 min at 50°C, followed by 10 min at 95°C and finally 40 cycles of 30 s at 95°C and 30 s at 60°C. The primers used are listed in Appendix Table A3. The amplicon expression was normalized to β‐actin in each sample.

### 2.6 Protein ELISAs

The hepatoblasts were treated with oestradiol (1, 10, and 100 nM) or 10 nM oestradiol + 40 µM DAPT for 24 h. Mice albumin and alpha‐fetoprotein levels in the cell‐free extracts were confirmed by using ELISA kits from Elabscience Biotechnology Laboratories following the manufacturer's instructions. The ELISA plates were firstly coated with 100 µl/well of different cell‐free extracts for 1.5 h at 37°C. Then, the liquids were discarded, and the plates were coated with 100 µl/well of biotinylated antibody for 1.5 h at 37°C. After that, the plates were washed three times with 350 µl PBS for 30 s every time. The liquids were discarded, and the enzyme conjugates were added using 100 µl/well volume. The plates were incubated for 0.5 h at 37°C. The plates were then washed for five times before 90 µl/well of TMB was added and incubated for 15 min at 37°C. The reaction was stopped with 50 µl/well of stop solution. The absorbance was read by ELISA instrument at 450 nm.

### 2.7 Histological analysis

Since day 6 of pregnancy, mice were treated as follows: (A) Mice in the control group were left intact. (B) Oestradiol (0.6 mg/kg) was given daily to the mice in the oestradiol group. (C) Oestradiol (0.6 mg/kg) and DAPT (10 mg/kg) were simultaneously given daily to the mice in the Oestradiol + DAPT group. (D) Tamoxifen (0.4 mg/kg) was given daily to the mice in the tamoxifen group.

Liver samples of the foetal mice at ED14, ED18 and newborn mice were collected. Then, the liver tissues were dehydrated, cleared, wax‐impregnated, embedded, and sectioned at 4 μm. After baking and dewaxing, the paraffin‐embedded sections were stained with haematoxylin and eosin (H&E) and then observed under an optical microscope. More than 20 portal areas of different groups of liver samples were observed for bile duct‐like structures counting under an optical microscope.

### 2.8 Immunohistochemistry

For antigen retrieval, the sections were dewaxed and placed in the boiling repairing liquid (0.01 M sodium citrate buffer, pH = 6.0) for 10 min. Then, the sections were quenched with 3% H_2_O_2_, incubated for 15 min at room temperature and blocked with 10% goat serum for 30 min at room temperature. The above sections were then respectively incubated with the primary antibodies overnight at 4°C. The secondary antibody, a goat anti‐rabbit IgG (diluted 1:200; Boster) was used with horseradish peroxidase (HRP)–streptavidin. The liver sections were observed under an optical microscope.

### 2.9 Western blot

The isolation of hepatoblasts is difficult and costly. However, Western blot has a great demand for hepatoblasts. After different treatments, expressions of hepatocyte lineage‐associated proteins, Notch1, Notch2 and Jagged1 in liver tissues were detected by Western blot analysis. EP tubes filled with the minced liver tissues were kept on ice for 30 min in cell lysis buffer with phenylmethanesulfonyl fluoride (PMSF) and phosphatase inhibitors. Samples containing 40 μg of protein were separated by 10% SDS‐PAGE. Then, the proteins were transferred to a PVDF membrane (Millipore) after electrophoresis for Western blot analysis using the primary antibodies and a 1:50,000 dilution of HRP‐conjugated secondary antibody. Proteins were then detected by ECL. The relative protein levels were quantified with ImageJ software.

### 2.10 Data analysis

All experiments were repeated three times. All data were presented as the mean ± standard deviations. The data were analysed with the Statistical Package for the Social Sciences (SPSS) software (V.13.0, SPSS Inc.). The measurement data were compared with single‐factor analysis of variance (one‐way ANOVA). *p* < 0.05 was considered statistically significant.

## 3 RESULTS

### 3.1 Oestradiol enhances hepatoblast differentiation into intrahepatic cholangiocytes via Notch signalling pathway

**FIGURE 1:**
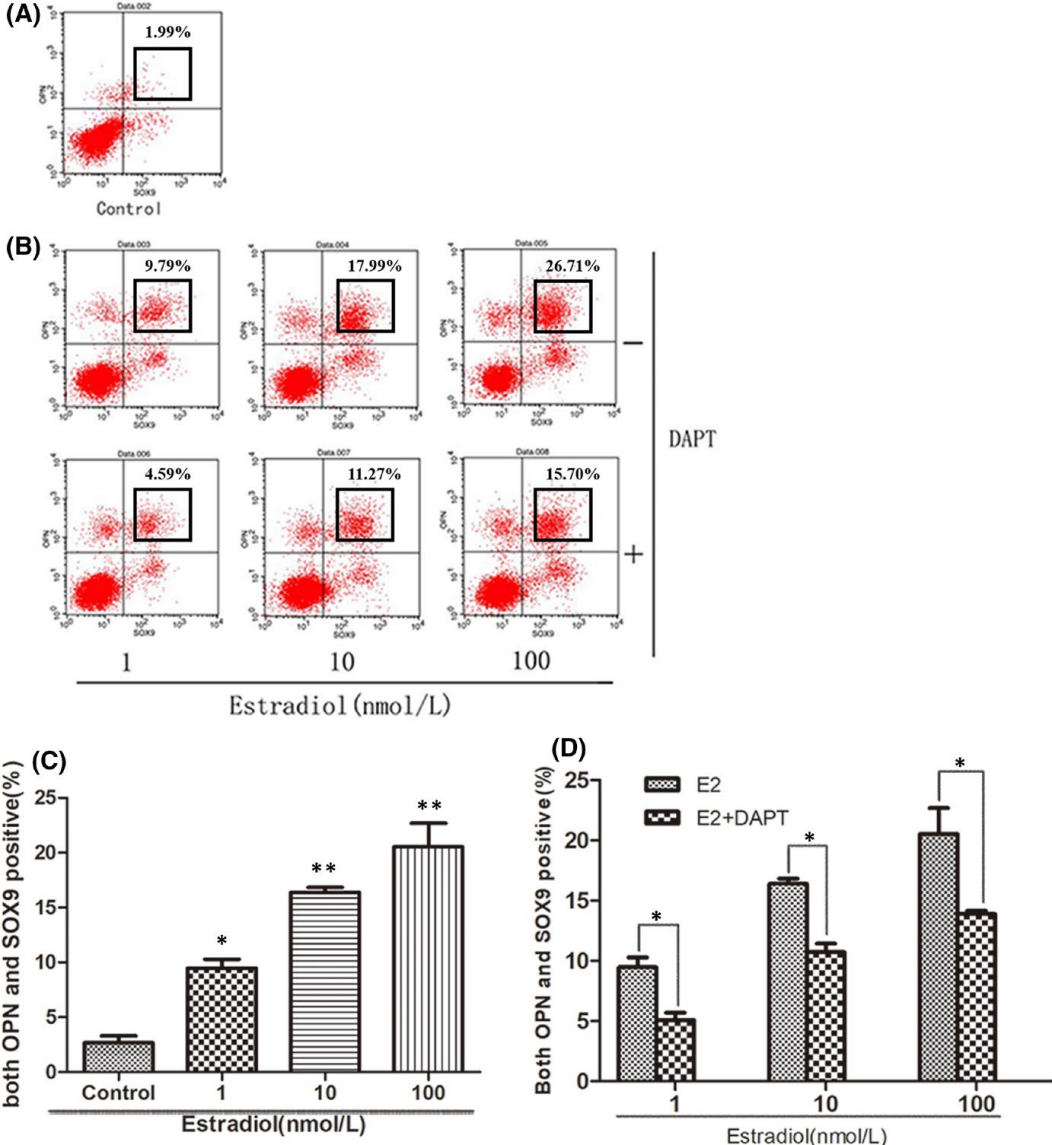
The effect of estradiol on the intrahepatic bile duct development in vivo. Liver samples of the fetal mice at ED14, ED18 and newborn mice were collected. (A–C) cholangiocyte‐lineage associated proteins were analyzed by immunohistochemical analysis, compared with the control group. The estradiol+ DAPT group was compared with the estradiol group. Scale bar, 25 µm (D–F) The mRNA expressions were measured relative to β‐actin by RT‐PCR, compared with the control group. The estradiol+ DAPT group was compared with the estradiol group. The levels of the hepatocyte‐lineage associated proteins were analyzed by western blot (G), including tyrosine aminotransferase (TAT) (H), tryptophan 2,3‐dioxygenase (TDO) (I), carbamoyl‐phosphate synthetase I (CPS‐I) (J) and cytochrome P450 (Cyt‐P450) (K). The estradiol group and tamoxifen group were compared with the control group. The estradiol+ DAPT group was compared with the estradiol group. (L) Liver samples of the newborn mice in different groups were collected for HE staining and bile duct‐like structures counting. The black arrows showed the intrahepatic bile ducts. Scale bar, 50 µm. (*, #*p* < 0.05, **, ##*p* < 0.01)

For measuring the differentiation of hepatoblasts, flow cytometer was used. Intrahepatic cholangiocytes were defined as Sox9^+^/OPN^+^ cells. As shown in Figure 1A,B, the intrahepatic cholangiocytes, respectively, make up 1.99%, 9.79%, 17.99% and 26.71% of the total in different oestradiol treated groups (0, 1, 10 and 100 nM). The percentages fell to 4.59%, 11.27% and 15.70% in oestradiol (1, 10, and 100 nM) + DAPT (40 μM) groups (*x*‐axis: Sox9, *y*‐axis: OPN). Oestradiol enhanced the proportion of intrahepatic cholangiocytes, compared with that in the control group (Figure 1C) (*p* < 0.05 or *p* < 0.01). The promotion of oestradiol was inhibited with the addition of DAPT (Figure 1D) (all *p* < 0.05).

**FIGURE 2:**
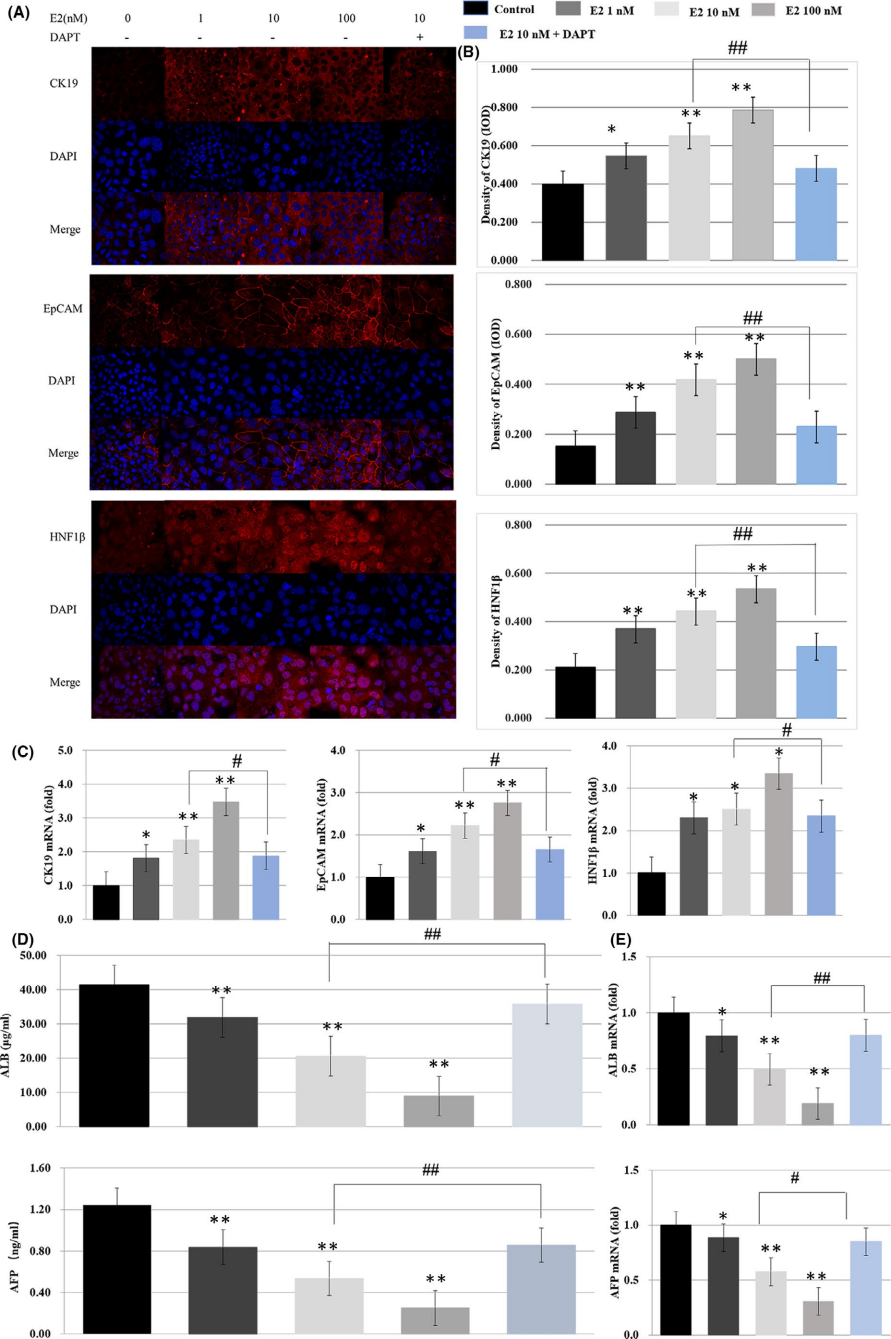
The effect of oestradiol on the cholangiocyte lineage and hepatocyte lineage‐associated proteins and genes in vitro. (A) The hepatoblasts were, respectively, incubated with anti‐CK19 antibody, anti‐EpCAM antibody, anti‐HNF1β antibody (red), and nuclei were stained with DAPI (blue). Scale bar, 10 µm. (B) Fluorescence quantification analysis was performed by comparing the IOD/pixel value of the protein in the hepatoblasts with the control group. The value in the 10 nM oestradiol + 40 µM DAPT group was compared with the 10 nM oestradiol group. (C) The mRNA expressions in the oestradiol groups were measured relative to β‐Actin by RT‐PCR, compared with the control group. The mRNA expressions in the 10 nM oestradiol + 40 µM DAPT group were compared with the 10 nM oestradiol group. (D) ELISA results of the ALB and AFP concentrations in the hepatoblast were compared with the control group. The results of the 10 nM oestradiol + 40 µM DAPT group were compared with the 10 nM oestradiol group. (E) The mRNA expressions of ALB and AFP in the oestradiol groups were compared with the control group by RT‐PCR. The mRNA expressions in the 10 nM oestradiol + 40 µM DAPT group were compared with the 10 nM oestradiol group (*, ^#^ *p* < 0.05, **, ^##^ *p* < 0.01).

As shown in Figure 2A,B, the immunofluorescence staining intensity for the cholangiocyte lineage‐associated proteins significantly increased following treatment with oestradiol in vitro, compared with the control group (*p* < 0.05 or *p* < 0.01). The intensity was markedly decreased following the treatment of 10 nM oestradiol + 40 µM DAPT, compared with the group treated with 10 nM oestradiol alone (*p* < 0.01). ELISA results showed that the hepatocyte lineage‐associated proteins decreased following treatment with oestradiol in vitro, compared with the control group (*p* < 0.05 or *p* < 0.01) (Figure 2D). RT‐PCR analysis confirmed the immunofluorescence staining and ELISA results (Figure 2C,E).

The above results demonstrated that oestradiol could promote the hepatoblast differentiation into intrahepatic cholangiocytes via Notch signalling pathway.

### 3.2 Oestradiol promotes the expression of Notch signalling pathway‐associated proteins in vitro

**FIGURE 3:**
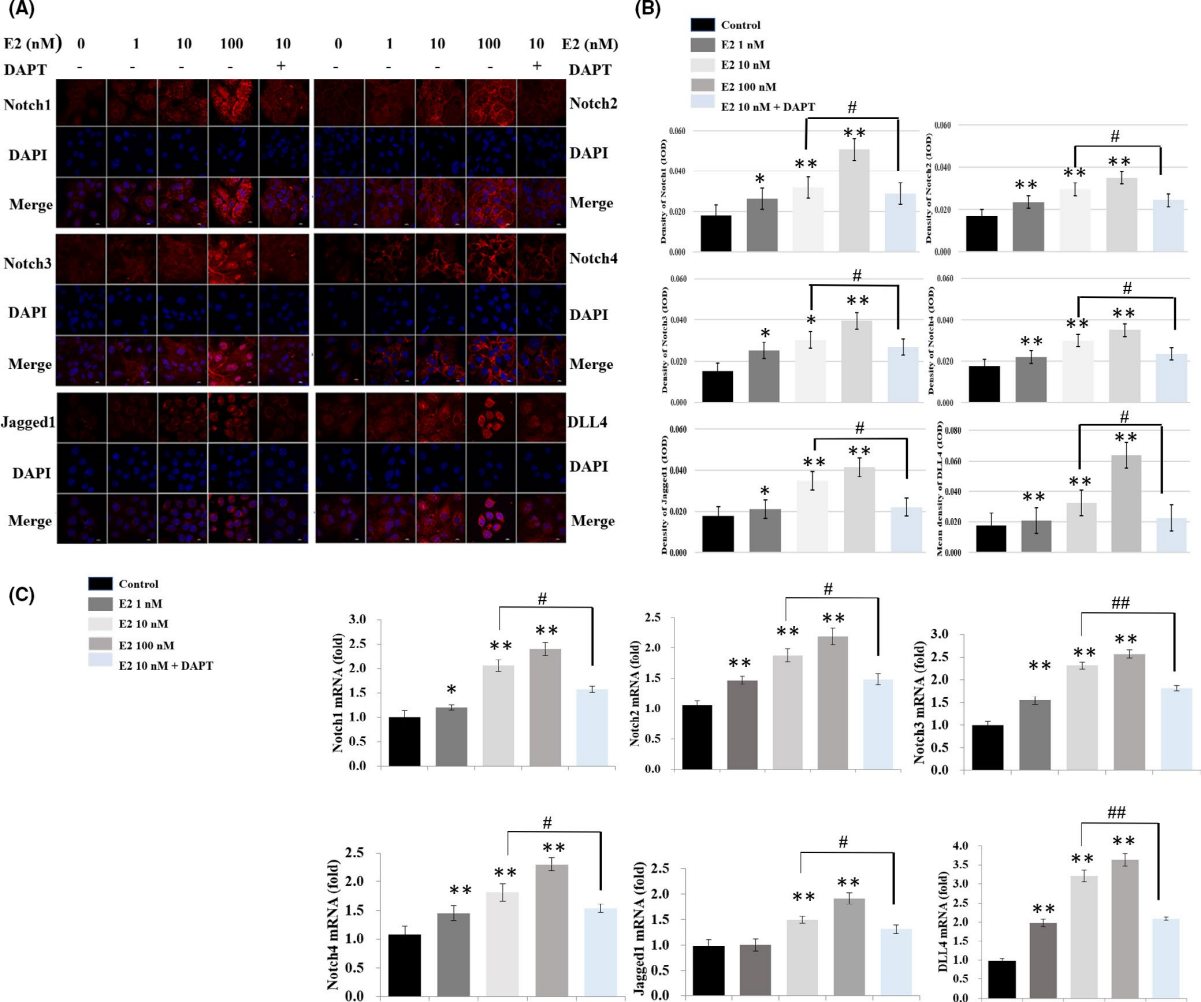
The effect of oestradiol on the Notch signalling pathway‐associated proteins and genes in vitro. (A) The hepatoblast was, respectively, incubated with anti‐Notch1 antibody, anti‐Notch2 antibody, anti‐Notch3 antibody, anti‐Notch4 antibody, anti‐Jagged1 antibody, anti‐DLL4 antibody (red), and nuclei were stained with DAPI (blue). Scale bar, 10 µm. (B) Fluorescence quantification analysis was performed by comparing the IOD/pixel value of the protein in the hepatoblast with the control group. The value in the 10 nM oestradiol + 40 µM DAPT group was compared with the 10 nM oestradiol group. (C) The mRNA expressions in the oestradiol groups were measured relative to β‐Actin by RT‐PCR, compared with the control group. The mRNA expressions in the 10 nM oestradiol + 40 µM DAPT group were compared with the 10 nM oestradiol group (*,^#^ *p* < 0.05, **, ^##^ *p* < 0.01)

**FIGURE 4:**
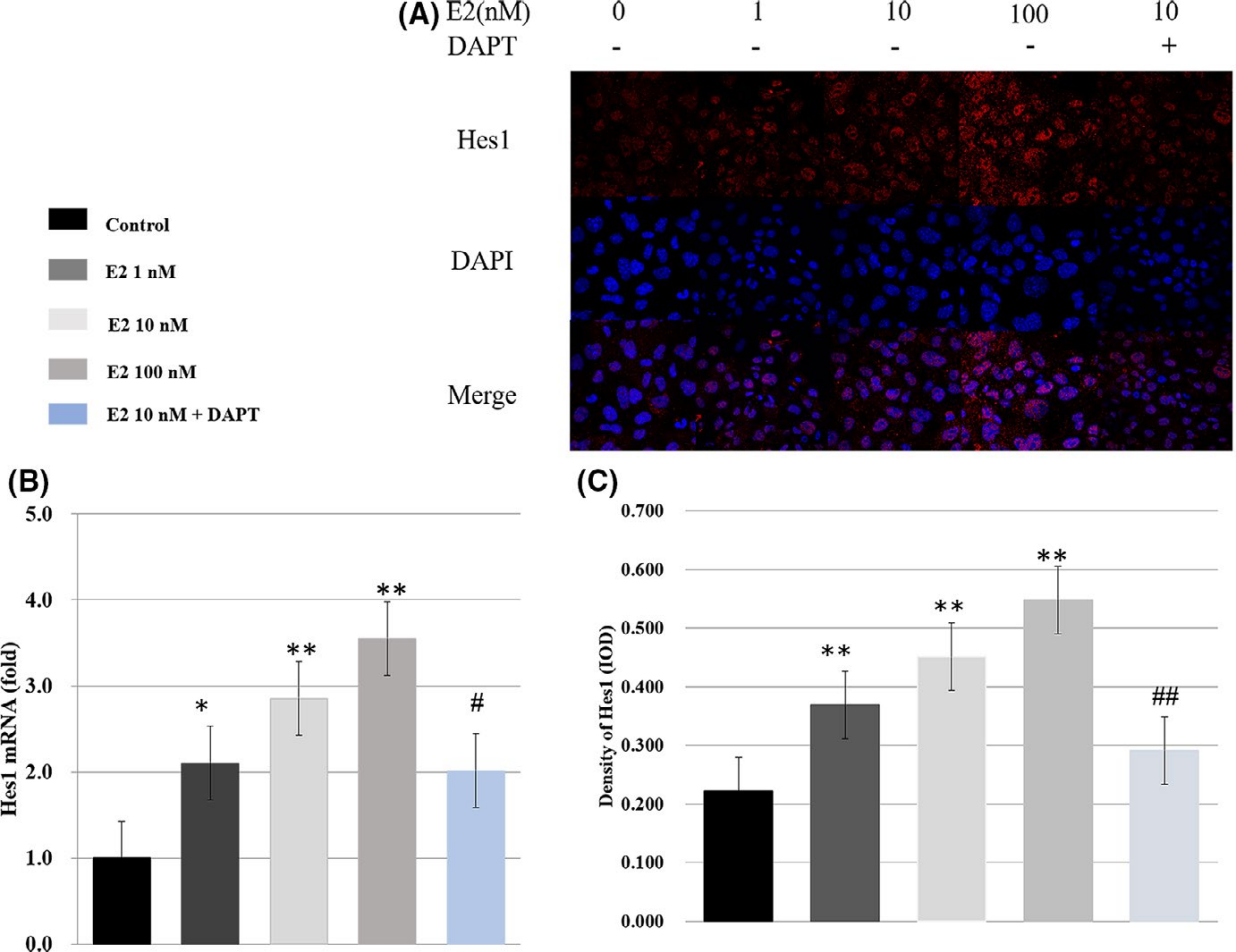
The effect of oestradiol on Hes1 in vitro. After different treatment, the hepatoblast was collected. (A, C) Hes1 in the hepatoblast was analysed by immunohistochemical analysis, compared with the control group. Results of the oestradiol + DAPT group were compared with the oestradiol group. Scale bar, 25 µm (B) The mRNA expression was measured relative to β‐actin by RT‐PCR, compared with the control group. The mRNA expression in the oestradiol + DAPT group was compared with the oestradiol group (*,^#^ *p* < 0.05, **, ^##^ *p* < 0.01)

As shown in Figures 3A,B and 4A,C, the immunofluorescence staining intensity for the receptors (Notch1, Notch2, Notch3 and Notch4), the ligands (JAG1 and DLL4) and Hes‐1 (the most important target genes of the pathway) increased significantly following treatment with oestradiol in the hepatoblasts, compared with the control group (*p* < 0.05 or *p* < 0.01). The staining intensity was markedly decreased following the treatment of 10 nM oestradiol + 40 µM DAPT, compared with the group treated with 10 nM oestradiol alone (*p* < 0.05).

RT‐PCR analysis confirmed the results of immunofluorescence staining (Figures 3C and 4B). Compared with the control group, the mRNA expression levels of Notch1, Notch2, Notch3, Notch4, JAG1, DLL4 and Hes‐1 were, respectively, increased (1.2‐, 1.5‐, 1.5‐, 1.4‐, 1.003‐, 2.1‐ and 2.104‐fold), (2.1‐, 1.9‐, 2.3‐, 1.7‐, 1.6‐, 2.1‐ and 2.853‐fold), (2.3‐, 2.0‐, 2.5‐, 2.4‐, 1.8‐, 3.6‐ and 3.547‐fold) following incubation with 1 nM, 10 nM and 100 nM oestradiol. The above results were compared with the control group (all *p* < 0.05 or *p* < 0.01). The mRNA expression levels were decreased following incubation with 10 nM oestradiol + 40 µM DAPT, compared with the group treated with 10 nM oestradiol alone (all *p* < 0.05 or *p* < 0.01).

The above results indicated that in vitro, oestradiol promotes the expression of Notch signalling pathway‐associated proteins.

### 3.3 Oestradiol promotes the expression of Notch signalling pathway‐associated proteins in vivo

In the in vivo experiment, we selectively detected the most important proteins relevant to intrahepatic bile duct development, namely Notch1, Notch2, Jagged1^21^ and Hes‐1.

**FIGURE 5:**
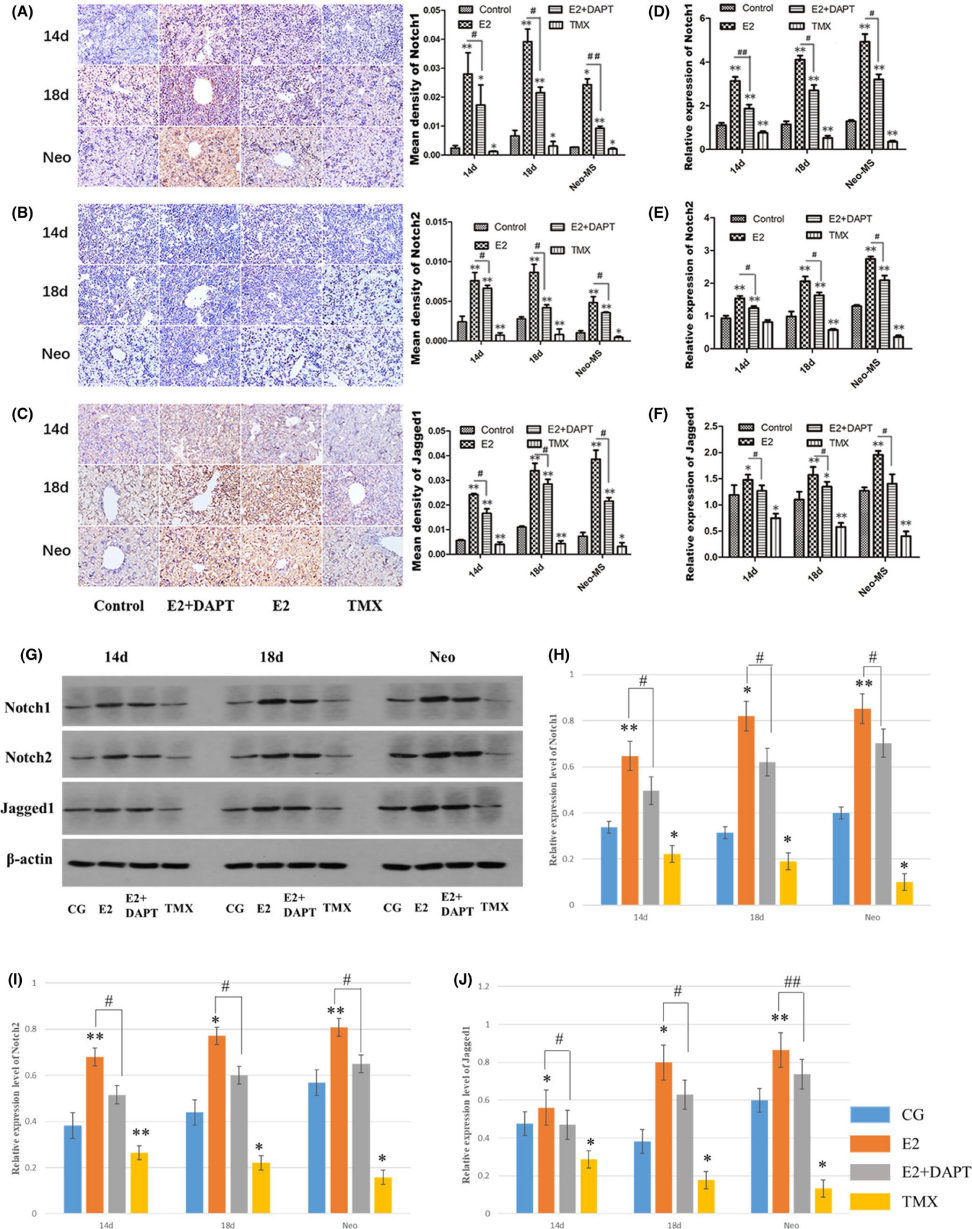
The effect of oestradiol on the Notch signalling pathway‐associated proteins and genes in the liver in vivo. Liver samples of the foetal mice at ED14, ED18 and newborn mice were collected. (A–C) Notch signalling pathway‐associated proteins in the hepatoblast were analysed by immunohistochemical analysis, compared with the control group. Results of the oestradiol + DAPT group were compared with the oestradiol group. Scale bar, 25 µm (D–F) The mRNA expressions were measured relative to β‐actin by RT‐PCR, compared with the control group. The mRNA expressions in the oestradiol + DAPT group were compared with the oestradiol group. (G–J) The levels of Notch signalling pathway‐associated proteins were analysed by Western blot. Results of the oestradiol group and tamoxifen group were compared with the control group. Results of the oestradiol + DAPT group were compared with the oestradiol group (*,^#^ *p* < 0.05, **, ^##^ *p* < 0.01).

**FIGURE 6:**
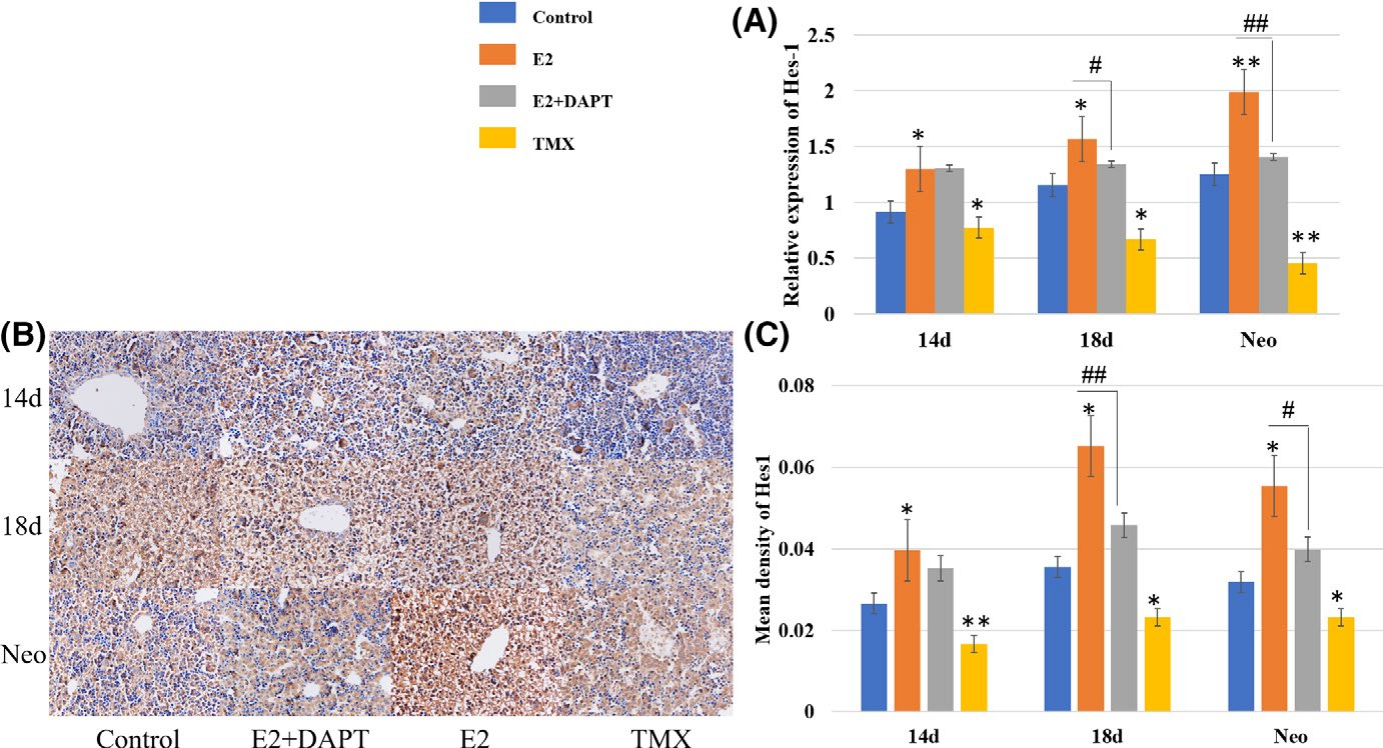
The effect of oestradiol on Hes1 in vivo. Liver samples of the foetal mice at ED14, ED18 and newborn mice were collected. (A) The mRNA expression was measured relative to β‐actin by RT‐PCR, compared with the control group. The oestradiol + DAPT group was compared with the oestradiol group. (B, C) The level of Hes1 was analysed by immunohistochemical analysis, compared with the control group. Results of the oestradiol + DAPT group were compared with the oestradiol group (*,^#^ *p* < 0.05, **, ^##^ *p* < 0.01) Scale bar, 25 µm

As shown in Figures 5A–C and 6B,C, in the liver samples of the foetal mice at ED14, ED18 and newborn mice, the immunohistochemical staining intensity for Notch1, Notch2, Jagged1 and Hes‐1 increased significantly in the oestradiol group, compared with that in the control group (*p* < 0.05 or *p* < 0.01). The intensity decreased obviously following the treatment with tamoxifen, an antioestrogenic drug, compared with that in the control group (*p* < 0.05 or *p* < 0.01). The intensity was decreased in the oestradiol + DAPT group, compared with the oestradiol group (*p* < 0.05 or *p* < 0.01), indicating that the oestradiol‐induced up‐regulation of these proteins was inhibited.

The results of immunohistochemical analysis were confirmed by RT‐PCR analysis (Figures 5D–F and 6A). Compared with the control group, the mRNA expression levels of Notch1, Notch2, Jagged1 and Hes‐1 in the oestradiol group were increased (2.7‐, 1.7‐ and 1.1‐fold), (2.6‐, 2.1‐ and 1.4‐fold), (3.8‐, 2.1‐ and 1.5‐fold), (1.3‐, 1.6‐ and 2.0‐fold) in the liver samples of the foetal mice at ED14, ED18 and newborn mice, respectively, compared with the control group (*p* < 0.05 or *p* < 0.01). The levels of the tamoxifen group were decreased to (69.2%, 88.0%, 62.9%), (45.7%, 58.4%, 52.6%), (27.3%, 28.2%, 31.8%), (77.6%, 66.5%, 45.2%), compared with the control group (*p* < 0.05 or *p* < 0.01). Compared with the oestradiol group, mRNA expression levels of the oestradiol + DAPT group decreased significantly (*p* < 0.05 or *p* < 0.01). Western blot analysis also confirmed the results of immunohistochemical and RT‐PCR (Figure 5G–J). These results clearly indicated that the expression of Notch signalling pathway‐associated proteins was promoted by oestradiol in vivo.

### 3.4 Oestradiol improves the intrahepatic bile duct development via Notch signalling pathway

**FIGURE 7:**
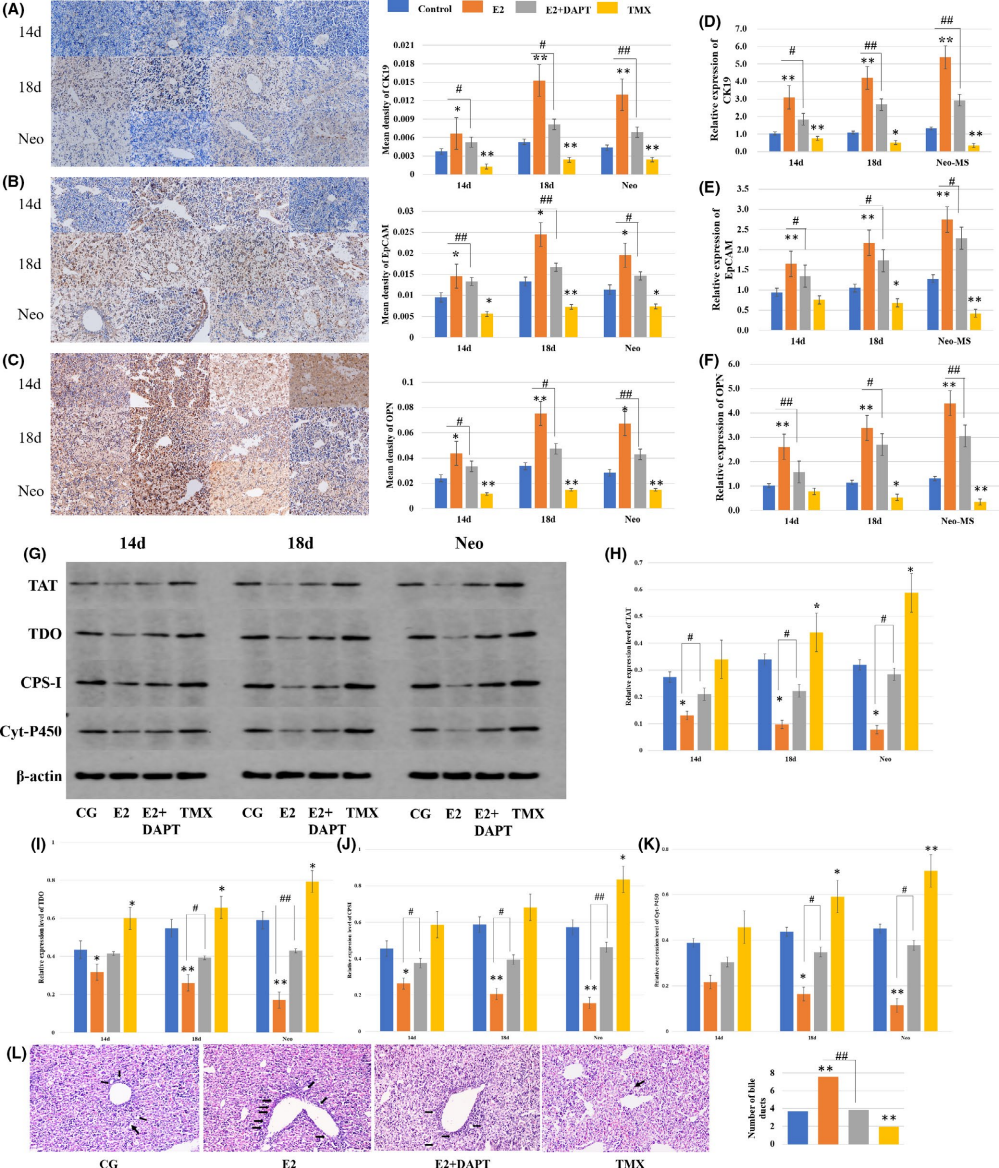
The effect of estradiol on the intrahepatic bile duct development in vivo. Liver samples of the fetal mice at ED14, ED18 and newborn mice were collected. (A–C) cholangiocyte‐lineage associated proteins were analyzed by immunohistochemical analysis, compared with the control group. The estradiol+ DAPT group was compared with the estradiol group. Scale bar, 25 µm (D–F) The mRNA expressions were measured relative to β‐actin by RT‐PCR, compared with the control group. The estradiol+ DAPT group was compared with the estradiol group. The levels of the hepatocyte‐lineage associated proteins were analyzed by western blot (G), including tyrosine aminotransferase (TAT) (H), tryptophan 2,3‐dioxygenase (TDO) (I), carbamoyl‐phosphate synthetase I (CPS‐I) (J) and cytochrome P450 (Cyt‐P450) (K). The estradiol group and tamoxifen group were compared with the control group. The estradiol+ DAPT group was compared with the estradiol group. (L) Liver samples of the newborn mice in different groups were collected for HE staining and bile duct‐like structures counting. The black arrows showed the intrahepatic bile ducts. Scale bar, 50 µm. (*, #*p* < 0.05, **, ##*p* < 0.01)

Liver samples of the newborn mice were collected for CK19, EpCAM and OPN staining (Figure 7A–C). The immunohistochemical staining intensity increased significantly in the oestradiol group, compared with that in the control group (*p* < 0.05 or *p* < 0.01). The intensity decreased obviously following the treatment with tamoxifen, compared with that in the control group (*p* < 0.05 or *p* < 0.01). The intensity was decreased in the oestradiol + DAPT group, compared with the oestradiol group (*p* < 0.05 or *p* < 0.01), indicating that the oestradiol‐induced up‐regulation of these proteins was inhibited.

The results of immunohistochemical analysis were confirmed by RT‐PCR analysis (Figure 7D–F). Compared with the control group, the mRNA expression levels of CK19, EpCAM and OPN in the oestradiol group were increased (3.1‐, 4.2‐ and 5.4‐fold), (1.6‐, 2.2‐ and 2.7‐fold), (2.6‐, 3.4‐ and 4.4‐fold) at ED14, ED18 and newborn mice, respectively, compared with the control group (*p* < 0.05 or *p* < 0.01). The levels of the tamoxifen group were decreased to (75.9%, 51.5%, 35.6%), (75.6%, 68.2%, 42.6%), (77.7%, 53.0%, 33.8%), compared with the control group (*p* < 0.05 or *p* < 0.01). Compared with the oestradiol group, mRNA expression levels of the oestradiol + DAPT group decreased significantly (*p* < 0.05 or *p* < 0.01).

As shown in Figure 7G–K, the data of hepatocyte maturation markers have also been assessed in in vivo study by Western blot analysis. It showed that the hepatocyte lineage‐associated proteins decreased following treatment with oestradiol, compared with the control group (*p* < 0.05 or *p* < 0.01).

## 4 DISCUSSION

Despite the recent advances in the understanding of the molecular pathogenesis of cholestasis liver diseases, some of the congenital biliary tract diseases remain incurable. Therefore, it is meaningful to identify factors influencing the bile duct development during embryogenesis.

Our study confirmed that the activation of the Notch signalling pathway promotes hepatoblast differentiation into intrahepatic cholangiocytes and development of intrahepatic bile ducts, which has been reported before.^6^, ^22^ Furthermore, in the present study, oestradiol was found to be an important factor promoting the differentiation of hepatoblasts into intrahepatic cholangiocytes and the intrahepatic bile duct development through the Notch signalling pathway during embryonic period.

Oestradiol is an important inducer of cell differentiation and growth. Oestradiol and oestradiol receptors (ER) act as the important roles in modulating the cholangiocyte proliferation. In liver, both the hepatocytes and the cholangiocytes have the ER. While the distribution of the receptors is different between them. The hepatocytes mainly express ER‐α, while the cholangiocytes express both ER‐β and ER‐α.^23^, ^24^, ^25^

Therefore, oestradiol first binds to oestrogen receptors and up‐regulates the relevant genes through the activation of oestrogen‐responsive elements (EREs), a specific nucleotide sequence. ERE, having the ability to confer oestradiol responsiveness, has been reported to be the crucial step for oestradiol activating the Notch signalling pathway.^13^, ^26^, ^27^

It has been proved that several extracellular signals, such as cytokines or hormones, affect the proliferation and differentiation of hepatoblasts. Dexamethasone (Dex) has been reported by lots of studies to change the differentiation and/or maturation of hepatoblasts.^28^, ^29^ It affects the hepatoblasts in combination with Oncostatin (OSM), a member of the interleukin 6‐related cytokine family.^30^ The combination induces the differentiation and maturation of hepatoblasts into hepatocytes, which is different with the promotion action of oestradiol in the present study.^31^, ^32^

Liver is known to be a target organ of oestradiol, which is crucial for liver regeneration after partial hepatectomy^33^ and liver growth in mammalian neonates.^34^ However, there are different opinions as to the mechanism of oestradiol promoting the liver growth. Uebi, T et al said that oestradiol controls the hepatocyte proliferation. While in Domenico et al.'s study, it has been found that oestradiol could be associated with the cholangiocytes proliferation.^35^ We agreed that oestradiol promotes the liver growth, but that the way was through promoting the differentiation of hepatoblasts into intrahepatic cholangiocytes and the development of intrahepatic bile ducts. In addition, we proposed that the activation of the Notch signalling is one of the key mechanisms of oestradiol in promoting the cholangiocytes proliferation besides ERK and PI3K/AKT.^11^, ^12^

Results from this study will also allow a better understanding of Alagille syndrome (ALGS), one of the important diseases characterized by congenital intrahepatic bile duct dysplasia. Approximately 70% of the ALGS patients required a liver transplantation during childhood.^36^ As an autosomal dominant disease caused by the mutation of Notch signalling ligand Jag1 or receptor Notch2, it has great heterogeneity with regard to the severity and clinical manifestations. In patients with ALGS and their families, some showed significant cholestasis and even cirrhosis, some were found disease‐free in spite of the same gene mutation.^37^, ^38^ Some scientists hypothesized that the heterogeneity is caused by a second candidate genetic modifier or some environmental factors.^36^ The results of our current study indicate that the level of oestradiol during the embryonic period is one of the important factors contributing to the heterogeneity.

In summary, this study indicates that oestradiol plays an important role in the hepatoblast differentiation into intrahepatic cholangiocytes and in the development of the intrahepatic bile ducts. Moreover, the mechanism may involve the Notch signalling pathway. Thus, based on our findings, regulating the oestradiol level during the embryo period may provide a new approach for the prevention and treatment of ductopenia. The regulation may even prevent Alagille syndrome (induce the disease‐free type). Of course, the effects of oestradiol on intrahepatic bile duct development of the Alagille syndrome mice need to be studied in the future.

## CONFLICT OF INTEREST

The authors confirm that there are no conflicts of interest.

## AUTHOR CONTRIBUTION

**Chen Dong:** Data curation (lead); Writing‐original draft (lead). **Xiao‐ping Luo:** Conceptualization (lead); Writing‐review & editing (lead). **Ben‐ping Zhang:** Data curation (lead); Investigation (equal); Writing‐review & editing (supporting). **Hong Wei:** Investigation (supporting); Writing‐review & editing (supporting). **Yan‐Qin Ying:** Methodology (equal); Writing‐review & editing (equal). **Ling Hou:** Methodology (equal); Writing‐review & editing (equal). **Wei Wu:** Data curation (equal); Writing‐review & editing (equal).

## Supporting information

Appendix S1

## Data Availability

No additional data are available.

## 1

**TABLE A1:** The reaction mix of first‐strand cDNA synthesis

Reagent	Volume (μl)
RNA	5 μg
Oligo (dT) 18 (10 μM)	2
dNTP (2.5 mM)	4
5×Hiscript Buffer	4
Hiscript Reverse Transcriptase	1
Ribonuclease Inhibitor	0.5
ddH_2_O (Rnase free)	Up to 20

**TABLE A2:** The reaction mix of qRT‐PCR

cDNA (diluted 1:8 with nucleotide/ RNAse free water)	4 μl
Forward Primer (10 μM)	0.4 μl
Reverse Primer (10 μM)	0.4 μl
SYBR Green Master Mix	10 μl
50×ROX Reference Dye 2	0.4 μl
H_2_O	4.8 μl

**TABLE A3:** Primer sequences used for real‐time polymerase chain reaction

Gene	Strand	Primer sequence (5′→3′)
β‐Actin	Sense	CACGATGGAGGGGCCGGACTCATC
	Antisense	TAAAGACCTCTATGCCAACACAGT
Notch1	Sense	GTGGACGGAGACAACTGGTA
	Antisense	TCCCCATGGTGATGCAAGAT
Notch2	Sense	CAGACTGGATGAACCGTGTGGAGA
	Antisense	GCTGCCTTTTGGGATAAGCTGGAAA
Notch3	Sense	CGATTCTCCTGTCGTTGTCTCCGTG
	Antisense	ACTTTGGCAGCTTTGACCCTGGTA
Notch4	Sense	GCAATGCCAAGGTCAGGAACACAGA
	Antisense	TCCAGCCCTCATCACACACACACAA
Jagged 1	Sense	GTGGACGGAGACAACTGGTA
	Antisense	TCCCCATGGTGATGCAAGAT
DLL4	Sense	GTCTGCAACTGTCCTTATGGCTTTG
	Antisense	GTCTGCAACTGTCCTTATGGCTTTG
CK19	Sense	GGCACGCTGGCAGAGACGGAG
	Antisense	TGGGGGTGGGCAGATTGTTGT
EpCAM	Sense	GAGTCCCTGTTCCATTCTTCTAA
	Antisense	GATCTCACCCATCTCCTTTATCT
HNF1β	Sense	ACAATCCCCAGCAATCTCAG
	Antisense	AGAGAACTGGACGGGCTGTA
ALB	Sense	TCTTCGTCTCCGGCTCTGCTTTTTC
	Antisense	TCGTTTCTTTCGGGCTCTTGTTTTG
AFP	Sense	GAATACTCAAGAACTCACCCCAACC
	Antisense	CCTCGTGTAACCAATAAGGAACAGA
HES1	Sense	CCGGCATTCCAAGCTAGAGA
	Antisense	CGTTGATCTGGGTCATGCAG
OPN	Sense	CACTCCAATCGTCCCTAC
	Antisense	CTTTCCGTTGTTGTCCTG
