# Supplementary material for: Oestradiol promotes the intrahepatic bile duct development of C57BL/6CrSlc mice during embryonic period via Notch signalling pathway
Source: J Cell Mol Med. 2021 Sep 8;25(19):9447–59. doi: 10.1111/jcmm.16888 (PMC8500961; doi:10.1111/jcmm.16888)
Supplement: Supplementary file 1 — Appendix S1 [file JCMM-25-9447-s001.docx]

| **Groups** | **Pictures (1,000 times)** | **area（sum）** | **IOD** | **Density (mean）** | **Density (mean）** |
| --- | --- | --- | --- | --- | --- |
|  |  |  |  |  |  |
| 1 | 1000-1_T001 | 381481 | 151620.72 | 0.39745 | 0.39891 |
|  | 1000-2_T001 | 583678 | 230450.24 | 0.39482 |  |
|  | 1000-3_T001 | 568301 | 229855.78 | 0.40446 |  |
| 2 | 1000-1_T001 | 402720 | 210289.05 | 0.52217 | 0.54507 |
|  | 1000-2_T001 | 320614 | 176455.37 | 0.55037 |  |
|  | 1000-3_T001 | 427285 | 240419.46 | 0.56267 |  |
| 3 | 1000-1_T001 | 393215 | 255382.38 | 0.64947 | 0.65029 |
|  | 1000-2_T001 | 410621 | 271969.41 | 0.66234 |  |
|  | 1000-3_T001 | 351621 | 224704.13 | 0.63905 |  |
| 4 | 1000-1_T001 | 334232 | 254704.40 | 0.76206 | 0.78569 |
|  | 1000-2_T001 | 326258 | 256491.48 | 0.78616 |  |
|  | 1000-3_T001 | 325578 | 263339.38 | 0.80884 |  |
| 5 | 1000-1_T001 | 390712 | 192251.55 | 0.49205 | 0.48096 |
|  | 1000-2_T001 | 265917 | 123506.17 | 0.46445 |  |
|  | 1000-3_T001 | 494404 | 240462.62 | 0.48637 |  |

The above is the raw immunofluorescence data of CK19

| **Groups** | **Pictures**  **(1,000 times)** | **area（sum）** | **IOD** | **Density (mean）** | **Density (mean）** |
| --- | --- | --- | --- | --- | --- |
|  |  |  |  |  |  |
| 1 | 1000-1_T001 | 265797 | 41964.49 | 0.15788 | 0.15043 |
|  | 1000-2_T001 | 273220 | 42772.71 | 0.15655 |  |
|  | 1000-3_T001 | 302813 | 41444.23 | 0.13686 |  |
| 2 | 1000-1_T001 | 235913 | 68605.02 | 0.29081 | 0.28688 |
|  | 1000-2_T001 | 279012 | 79586.62 | 0.28524 |  |
|  | 1000-3_T001 | 154765 | 44045.35 | 0.28460 |  |
| 3 | 1000-1_T001 | 102640 | 42666.37 | 0.41569 | 0.41736 |
|  | 1000-2_T001 | 125393 | 51628.16 | 0.41173 |  |
|  | 1000-3_T001 | 109377 | 46446.61 | 0.42465 |  |
| 4 | 1000-1_T001 | 119368 | 62497.07 | 0.52357 | 0.49949 |
|  | 1000-2_T001 | 265230 | 129928.31 | 0.48987 |  |
|  | 1000-3_T001 | 142044 | 68897.95 | 0.48505 |  |
| 5 | 1000-1_T001 | 160914 | 37214.89 | 0.23127 | 0.22927 |
|  | 1000-2_T001 | 133484 | 29900.81 | 0.22400 |  |
|  | 1000-3_T001 | 110281 | 25645.21 | 0.23254 |  |

The above is the raw immunofluorescence data of EPCAM.

| **Groups** | **Pictures (1,000 times)** | **area（sum）** | **IOD** | **Density (mean）** | **Density (mean）** |
| --- | --- | --- | --- | --- | --- |
|  |  |  |  |  |  |
| 1 | 1000-1_T001 | 434238 | 101008.62 | 0.23261 | 0.21047 |
|  | 1000-2_T001 | 521934 | 102229.58 | 0.19587 |  |
|  | 1000-3_T001 | 430731 | 87411.69 | 0.20294 |  |
| 2 | 1000-1_T001 | 455886 | 163494.32 | 0.35863 | 0.36784 |
|  | 1000-2_T001 | 493007 | 188923.27 | 0.38321 |  |
|  | 1000-3_T001 | 466500 | 168720.76 | 0.36167 |  |
| 3 | 1000-1_T001 | 410019 | 182009.28 | 0.44390 | 0.44167 |
|  | 1000-2_T001 | 418593 | 185296.71 | 0.44267 |  |
|  | 1000-3_T001 | 411586 | 180457.45 | 0.43844 |  |
| 4 | 1000-1_T001 | 430141 | 223918.44 | 0.52057 | 0.53365 |
|  | 1000-2_T001 | 445861 | 239275.38 | 0.53666 |  |
|  | 1000-3_T001 | 401162 | 218116.59 | 0.54371 |  |
| 5 | 1000-1_T001 | 510276 | 141064.77 | 0.27645 | 0.29511 |
|  | 1000-2_T001 | 434935 | 125889.72 | 0.28944 |  |
|  | 1000-3_T001 | 395346 | 126284.45 | 0.31943 |  |

The above is the raw immunofluorescence data of HNF1β.

The above is the raw immunofluorescence data of HES1.

| **Groups** | **Pictures (1,000 times)** | **area（sum）** | **IOD** | **Density (mean）** | **Density (mean）** |
| --- | --- | --- | --- | --- | --- |
|  |  |  |  |  |  |
| 1 | 1000-1_T001 | 241867 | 56179.35 | 0.23227 | 0.22250 |
|  | 1000-2_T001 | 245469 | 53100.23 | 0.21632 |  |
|  | 1000-3_T001 | 260306 | 56982.95 | 0.21891 |  |
| 2 | 1000-1_T001 | 227855 | 87962.08 | 0.38604 | 0.36856 |
|  | 1000-2_T001 | 186552 | 69984.35 | 0.37515 |  |
|  | 1000-3_T001 | 131078 | 45153.11 | 0.34448 |  |
| 3 | 1000-1_T001 | 154262 | 68282.21 | 0.44264 | 0.45107 |
|  | 1000-2_T001 | 127653 | 58977.83 | 0.46202 |  |
|  | 1000-3_T001 | 117516 | 52714.06 | 0.44857 |  |
| 4 | 1000-1_T001 | 109651 | 58425.64 | 0.53283 | 0.54829 |
|  | 1000-2_T001 | 128272 | 69569.87 | 0.54236 |  |
|  | 1000-3_T001 | 85153 | 48509.77 | 0.56968 |  |
| 5 | 1000-1_T001 | 99205 | 29339.51 | 0.29575 | 0.29157 |
|  | 1000-2_T001 | 82180 | 24622.34 | 0.29961 |  |
|  | 1000-3_T001 | 130767 | 36531.04 | 0.27936 |  |
